# The Effects of Compulsory Licensing: A Case Study of HIV Drugs

**DOI:** 10.1002/hec.70123

**Published:** 2026-06-23

**Authors:** Nicolau Martin‐Bassols, Miquel Serra‐Burriel, Kerstin Noelle Vokinger

**Affiliations:** ^1^ Center for Economic and Social Research (CESR) University of Southern California Los Angeles California USA; ^2^ Epidemiology Biostatistics and Prevention Institute University of Zurich Zürich Switzerland; ^3^ Faculty of Law University of Zurich and Department of Health Sciences and Technology Academic Chair for Regulation in Law Medicine, and Technology ETH Zurich Zürich Switzerland

**Keywords:** antiretrovirals, compulsory licensing, essential medicines, HIV, market access

## Abstract

Compulsory licensing is a legal mechanism that allows governments the use of patented medicines without the owner's consent, subject to conditions and compensation, to meet public‐health needs. Despite its policy relevance and increasing use, empirical evidence on its market effects remains limited. This study examines the association between compulsory licensing and the commercial accessibility and affordability of HIV medications across 11 countries from 2002 to 2022. We combine quarterly data on drug sales and prices from IQVIA with implementation dates from the TRIPS Flexibilities Database, focusing on 21 HIV drugs subject to at least one compulsory license. Using a staggered difference‐in‐differences design, we estimate the average treatment effect on the treated (ATT) of compulsory licensing on drug consumption and prices, under a parallel trends assumption. We find an economically meaningful increase in retail and hospital sales following the implementation of compulsory licenses, amounting to approximately 21% of a standard deviation in the countries that implemented them. In addition, we estimate a reduction in prices of approximately 19% of a standard deviation. These associations are consistent with compulsory licenses having contributed to improved availability of HIV drugs through commercial channels, with meaningful effects on prices. Our estimates do not capture—and therefore cannot rule out—potential additional effects on access via public importation or production, donations, or NGO‐led supply chains.

## 1 Introduction

Compulsory licenses are governmental authorizations that allow third parties to produce or process a patented product without the explicit consent of the patent holder. This concept is incorporated in the TRIPS (Trade‐Related Aspects of Intellectual Property Rights) Agreement, which is overseen by the World Trade Organization (WTO). Since its inception in January 1995, the TRIPS Agreement has included provisions allowing for compulsory licensing as one of the flexibilities within patent protection frameworks (World Trade Organization 2006).

Compulsory licensing *“is when a government allows someone else to produce a patented product or process without the consent of the patent owner or plans to use the patent‐protected invention itself for the domestic market”* (World Trade Organization 2006). The patent owner retains rights over the patent and is entitled to be paid compensation for the copies made under the compulsory license. Countries have autonomy in establishing the criteria for granting compulsory licenses and defining what constitutes a national emergency, thereby allowing them to tailor the application of compulsory licensing to their specific circumstances and needs (Smith et al. 2009).

The 2001 Doha Ministerial Conference clarified and reaffirmed the flexibility of the TRIPS Agreement regarding public health concerns. One of its key outcomes was the recognition of the sovereign right of governments to utilize compulsory licensing as a tool to address public health emergencies. The declaration underscores the need for access to affordable medicines, especially in developing countries (Correa 2002).

The HIV pandemic was an important driver for the outcomes of the 2001 Doha Ministerial Conference (World Trade Organization 2001). HIV was first documented by the US Centers for Disease Control in 1981, and at the time was considered a near‐certain death sentence. The landscape of HIV treatment began to shift with the introduction of effective antiretroviral therapy. This transformation started in 1987 with the introduction of azidothymidine (Tseng et al. 2015), and reached a crucial milestone with the approval of highly active antiretroviral therapy (HAART). HAART, comprising a three‐drug regimen, marked a new era in HIV treatment, transforming it from a deadly disease to a chronic yet manageable health condition.

Despite the remarkable success in the R&D process of antiretroviral therapy policies, a significant gap persisted in access to treatment. In 2002, only 2.7% of the estimated 11 million eligible adults were administered antiretroviral therapy, prompting urgent calls for action. There was a rapid transformation in the following years, which resulted in 47% of eligible individuals receiving treatment between 2004 and 2010 (UNAIDS 2011a). Various factors contributed to this surge in antiretroviral treatment rates, including reductions in drug prices and concerted efforts by countries and civil society. Compulsory licenses have been credited as a pivotal factor contributing to the increased consumption of antiretrovirals and access to treatment (UNAIDS 2011b). However, despite these assertions, there remains a notable absence of empirical evidence substantiating these claims.

A few case studies documented the implementation of compulsory licenses for various HIV drugs in different countries. These studies describe negotiated prices before and after the issuance of compulsory licenses, as well as changes in consumption patterns. Examples include compulsory licenses for drugs such as Zidovudine, Lamivudine (150 mg pill), Saquinavir (200 mg capsule), and Ritonavir in Brazil (Saroha et al. 2015), as well as Efavirenz in Thailand (Steinbrook 2007). There is one review article (Urias and Ramani 2020) comparing pre‐ and post‐compulsory licensing prices using non‐standardized data sources, which suggests a potential price‐lowering effect. While these papers provide suggestive evidence of the potential effectiveness of compulsory licenses in improving access to treatments, they omit counterfactuals in their analysis, and hence lack the credibility required for a causal impact assessment.

Another group of prior studies constructed models and forecast the anticipated effect of compulsory licenses on the health system (Bond and Saggi 2014; Morin et al. 2022; Stavropoulou and Valletti 2015). However, none of these papers empirically validated their predictions. In contrast, there is now credible evidence that voluntary licensing agreements for hepatitis C drugs did in fact result in increased access (Simmons et al. 2019).

The objective of the present study is to address the literature gap on the effects of compulsory licenses by combining a rigorous quasi‐experimental design with comprehensive data on the global HIV treatment market.

## 2 Methods

This study comprises an empirical analysis of HIV drug sales and pricing dynamics before and after the implementation of compulsory licenses in 11 countries (Brazil, Canada, China, Ecuador, Germany, Indonesia, Malaysia, Pakistan, Philippines, Russia, Thailand).

### 2.1 Data

We used data from the TRIPS Flexibilities Database from the Medicines Law & Policy project (Medicines Law and Policy 2024). The database contains publicly available information on countries, dates, products, patent status, and execution of compulsory licenses. Our sample was restricted to implemented compulsory licenses of HIV drugs (69% of all compulsory licenses).

We matched this database with drug consumption and pricing data from IQVIA (IMS Health) spanning from 2002 to 2022 for 72 countries (11 “treated” and 61 “controls”). IQVIA records commercial drug transactions through formal retail and hospital channels; it does not capture procurement through centralized government tenders, donor‐funded programmes (e.g., PEPFAR, Global Fund), or non‐governmental distribution networks, which may represent a substantial share of antiretroviral procurement in lower‐income settings. We return to this limitation in the Discussion.

The dataset contains quarterly information on list prices, sales volumes, companies, molecules, doses, and patent status for sold drugs. The quantity variable is expressed in IQVIA standard units, where one standard unit corresponds to the smallest separately administrable dose of a product (e.g., one tablet, one capsule, or one defined volume of liquid). Price is the observed list price per standard unit. For the included countries, both retail and hospital sales data were available. Our unit of observation was the molecule–country pair, observed at the quarterly level, and our outcomes were HIV drug standard units consumed and HIV drug list prices per standard unit in a given quarter. A “molecule” is defined as a distinct active pharmaceutical ingredient (API) or, in the case of fixed‐dose combinations (FDCs), the set of APIs co‐formulated in a single product. FDCs are recorded as separate entries from their individual components, reflecting the fact that the compulsory license in each case was granted at the product level. Sensitivity analyses restricting to single‐ingredient molecules are reported in the Supporting Information S1.

The IQVIA dataset over‐represents developed countries, in contrast with compulsory licenses, which were predominantly implemented in developing countries. Supporting Information S1: Table S1 reports summary statistics for the 39 treated country‐drug units separately.

**FIGURE 1:**
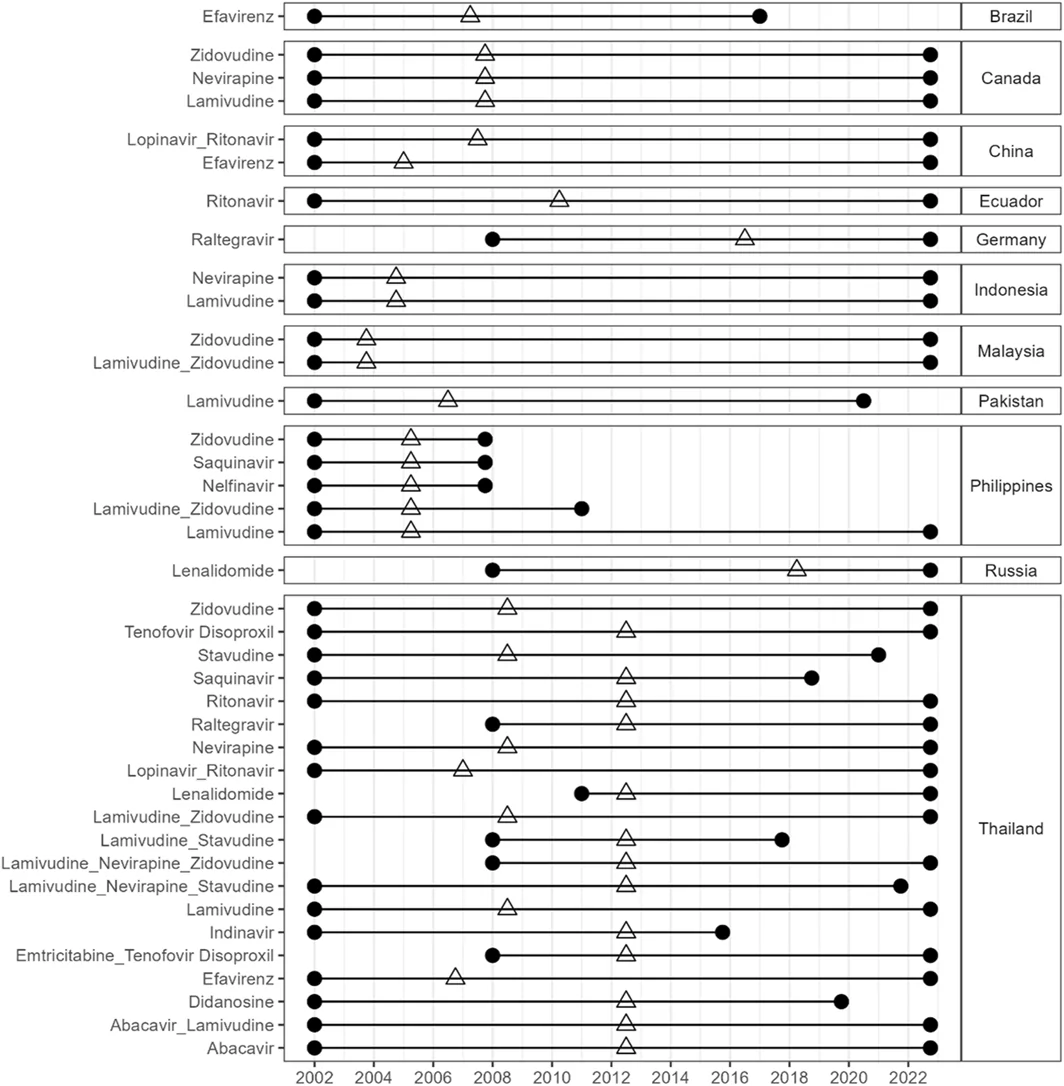
Observed IQVIA drug consumption and compulsory‐license implementation by country and date. The time unit of estimation is at the molecule–quarter level. Dots mark the first and last quarters in which IQVIA records positive sales for that molecule–country pair; gaps within the series reflect quarters of zero reported sales, which may indicate market withdrawal, IQVIA data gaps, or procurement shifts outside commercial channels. Triangles mark the implementation date of the compulsory license. *Source:* IQVIA and TRIPS Flexibilities Database.

Our analytical sample comprised 21 distinct HIV drugs, which were affected by at least one implemented compulsory license and consumed by at least one “treated” country from 2002 to 2022 (Figure 1). In Figure 1, some drug–country series have gaps or end before 2022. These reflect periods of zero reported sales in the IQVIA data, which may indicate market withdrawal, data unavailability, or procurement shifts outside commercial channels; they do not necessarily represent complete absence of the drug from the market. Our estimator accommodates an unbalanced panel. The “treatment” group was classified as country‐drug involved in compulsory licenses, while those that did not implement any compulsory license were classified as the “control” group.

### 2.2 Statistical Analysis

To assess the effect of compulsory licenses on the consumption and pricing dynamics of HIV drugs, we leveraged the timing of compulsory licenses across countries from 2002 to 2022. We used quasi‐experimental variation created by the staggered implementation of compulsory licenses, which under a set of identifying assumptions allows us to estimate the conditional association between compulsory license implementation and drug consumption and prices with a difference‐in‐differences strategy.

Identification of the ATT requires two main assumptions. First, *parallel trends conditional on fixed effects*: in the absence of a compulsory license, the trajectory of sales and prices for treated units would have evolved in parallel to those of control units, conditional on country‐drug and calendar‐time fixed effects. Second, *no interference*: the compulsory license issued for a given drug in a given country does not affect prices or quantities in control units. We assess the plausibility of both assumptions empirically and through sensitivity analyses described below. We note that compulsory licenses are not randomly assigned and may be triggered by budget pressures, negotiation breakdowns, or public‐health crises. Our identifying assumptions therefore require that, conditional on the fixed effects, these time‐varying factors do not differentially drive pre‐treatment trends.

The main assumption of our empirical strategy was that in the absence of a compulsory license implementation, the trajectory of sales and prices for the “treated” units would have evolved in parallel to the “control” ones. Under this assumption, our model rules out that results are driven by constant differences across drugs and common national consumption and pricing trends. To provide empirical support for this assumption, we estimated a dynamic version to examine the existence of pre‐trends with the Callaway and Sant’Anna difference‐in‐difference estimator (Callaway and Sant’Anna 2021). Another reason to use the estimator instead of a standard two‐way fixed‐effects model is that should the treatment effects be heterogeneous across time, the estimator would be inconsistent. The regression specification is as follows:

$$Y_{\mathrm{cdt}}=\sum_{\tau =-k}^{K}\alpha_{\tau }\cdot 1\;\left[\tau =t-G_{cd}\right]+\mu_{cd}+\delta_{t}+\varepsilon_{\mathrm{cdt}}$$

where $1\left[\tau =t-G_{cd}\right]$ is an indicator equal to one if quarter t is τ quarters away from the treatment quarter $G_{cd}$. The subscripts c, d, and t index country, drug, and calendar quarter respectively denotes the quarter‐of‐year implied by t. The coefficients $\alpha_{\tau }$ capture the estimated association of the compulsory license at each event time τ. Country‐drug fixed effects $\mu_{cd}$ control for time‐invariant differences in treatment regimes, drug composition, and baseline access levels. Calendar‐time fixed effects $\delta_{t}$ are included at the quarter‐year level and account for global trends, shocks, and seasonality common to all countries in each calendar quarter. The error term $\varepsilon_{\mathrm{cdt}}$ is clustered at the country‐drug level.

To address potential time‐varying confounding, we include the following covariates in the outcome regression: log GDP per capita (PPP), log population (World Bank WDI), HIV prevalence among adults aged 15–49 (Hay et al. 2025), and drug age (years since first regulatory approval). The estimator used is the regression‐adjusted Callaway–Sant’ Anna estimator, which fits an outcome regression exclusively on control units and uses the predicted counterfactual to estimate the ATT. Because the outcome regression is fitted on control units spanning all 61 countries and all different molecules, drug age varies meaningfully in the estimation sample (approvals ranging from 1987 to 2007). The doubly‐robust propensity score variant is not used: because 20 of 39 licenses are in Thailand, many treatment cohorts consist entirely of Thai units sharing identical country‐level covariate values at any base period, rendering the propensity score design matrix rank‐deficient. WHO EML status and MPP license status are also excluded: these binary indicators have near‐zero variance among control units across most (g,t) comparisons, as most ARVs were already EML‐listed before the observation window and most molecules have no MPP license throughout.

The target estimand was the average treatment effect on the treated (ATT), defined as:

$$\text{ATT}\left(g,t\right)=E\left[Y_{\mathrm{cdt}}\left(1\right)-Y_{\mathrm{cdt}}\left(0\right)|G_{cd}=g,\;t\geq g\right]$$

where $G_{cd}$ indicates the treatment onset for the country‐drug pair, and t indexes post‐treatment periods. Standard errors were clustered at country‐drug level and bootstrapped using 5000 draws with replacement. The outcome variables were standard units sales and prices. Both were standardised and centered at the country‐ATC‐1 level. Given that over half of the licenses (20/39) were enacted in Thailand, we estimated the effects specifically for the Thai market using the same analytical approach.

To formally assess the parallel trends assumption, we report bootstrap 95% confidence intervals for all pre‐treatment event‐study coefficients from the main specification. This approach uses the same bootstrap standard errors as the main results. If all pre‐treatment CIs include zero the data are consistent with the parallel trends assumption; we additionally report the ratio of the maximum absolute pre‐treatment coefficient to the aggregate post‐treatment ATT as a measure of the practical magnitude of any pre‐trend.

Many compulsory licenses are preceded by lengthy negotiations or public announcements that may trigger price concessions before formal implementation. We assess this through a sensitivity analysis using an anticipation window of four quarters prior to the recorded implementation date (Supporting Information S1: Figure S6).

We report the following pre‐specified sensitivity analyses in the Supporting Information S1: (i) restricting to high‐quality IQVIA countries (excluding Ecuador, Malaysia, Pakistan); (ii) not‐yet‐treated control group; (iii) single‐ingredient molecules only; (iv) excluding therapeutic substitutes; (v) same‐molecule control group; (vi) log‐transformed outcomes; (vii) no covariates.

## 3 Results

We analyzed 39 compulsory licenses across 21 HIV medications in 11 countries. We find broad support for the parallel trends assumption: bootstrap 95% confidence intervals for pre‐treatment event‐study coefficients include zero in 28 of 31 assessed periods for consumption (90.3%) and 29 of 31 periods for prices (93.5%), with the coefficients that do exclude zero being small in magnitude (maximum absolute value 0.11 SD for consumption and 0.02 SD for prices, both well below the estimated post‐treatment effects). Full pre‐treatment coefficient estimates and confidence intervals are reported in Supporting Information S1: Table S2.

**FIGURE 2:**
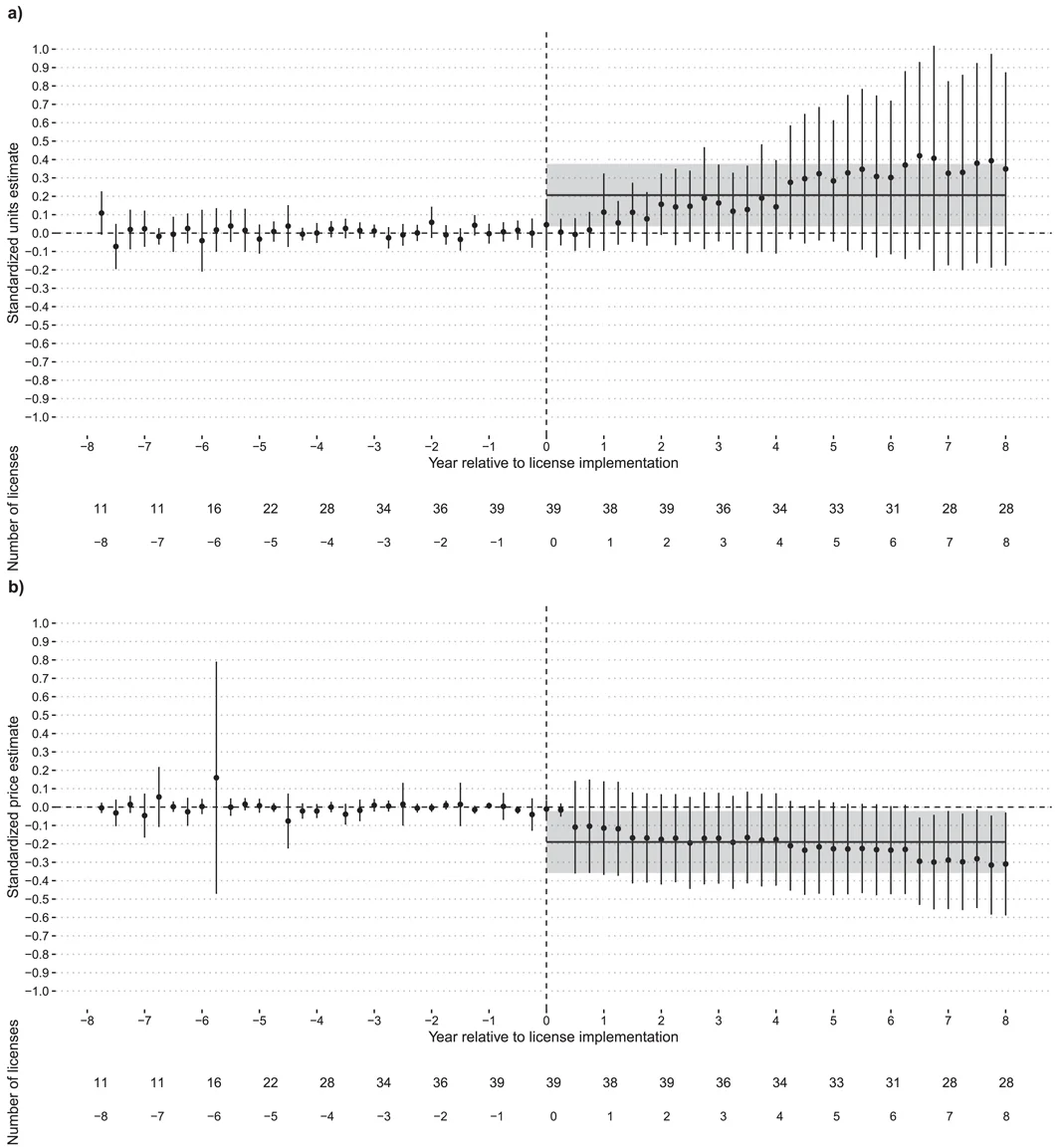
Event study estimates of drug‐unit consumption and prices before and after compulsory license implementation. Approximately 60,900 observations after dropping rows with missing covariate values (3.9% of the sample). The control group is never treated. Outcomes are standardised with mean zero and standard deviation one at the country‐ATC‐1 level. The regression‐adjusted Callaway–Sant’Anna estimator is used with covariates: log GDP per capita (PPP), log population, HIV prevalence (GBD 2023), and drug age (years since first regulatory approval). Clustered bootstrapped standard errors were computed at the molecule–country level through 5000 repetitions and the resulting 95% confidence intervals are reported. The dotted vertical line marks the moment of compulsory license implementation. The solid horizontal line depicts the aggregated ATT, while the shaded area represents its 95% confidence interval. Estimates are conditional on the parallel trends and no‐interference assumptions.

Figure 2 presents both dynamic and aggregated ATT estimates, illustrating the estimated association between compulsory licensing and HIV drug consumption and pricing. All estimates are conditional on the parallel trends and no‐interference assumptions described in the Methods and should be interpreted causally only under these identifying assumptions.

Panel (a) of Figure 2 displays the dynamic ATT estimates for standardised units of HIV drug consumption. Post‐implementation, we observe a gradual upward trend in consumption, reaching approximately 40% of a standard deviation 8 years after the license was issued. While the dynamic estimates exhibit wide confidence intervals—limiting precision—the aggregated ATT is more informative, estimated at 0.21 (95% CI: 0.04–0.38). These results suggest a statistically significant and economically meaningful estimated association between compulsory licensing and drug consumption.

Panel (b) of Figure 2 presents results for standardised HIV drug prices. Following compulsory license implementation, prices show a consistent decline over time, with dynamic ATT estimates indicating a reduction between 10% and 20% of a standard deviation. The aggregated ATT is −0.19 (95% CI: −0.36 to −0.02), suggesting a statistically significant and economically meaningful estimated decrease in prices of about 19% of a standard deviation.

These patterns remain broadly robust across alternative specifications (Supporting Information S1: Table S3). The consumption effect is statistically significant across most sensitivity analyses, and the price effect is similarly robust. Results are qualitatively unchanged when excluding lower‐quality data from Ecuador, Malaysia, and Pakistan (Supporting Information S1: Figure S1), when including not‐yet‐treated units in the control group (Supporting Information S1: Figure S2), when restricting to the same‐molecule control group (Supporting Information S1: Figure S5), when excluding fixed‐dose combinations (Supporting Information S1: Figure S7), when excluding therapeutic substitutes (Supporting Information S1: Figure S8), when using log‐transformed outcomes (Supporting Information S1: Figure S9), and when re‐estimating without covariates (Supporting Information S1: Figure S10). The anticipation sensitivity analysis (Supporting Information S1: Figure S6) yields estimates of similar magnitude.

When analyzing the Thai market, which implemented over half of the HIV licenses in our sample, we find stronger estimated effects: an increase of 0.36 SD (95% CI: 0.09–0.64) in quantities, and a 0.22 SD (95% CI: −0.54 to 0.09) decrease in prices (Supporting Information S1: Figure S3). Among non‐Thai countries, the estimated consumption effect is smaller (0.10 SD, 95% CI: −0.08 to 0.27) and statistically imprecise, consistent with Thailand driving a substantial share of the pooled result (Supporting Information S1: Figure S4).

## 4 Discussion

Our results indicate that compulsory licensing is associated with a tangible estimated rise in consumption, and with a statistically significant estimated decline in prices. These associations are economically meaningful and directionally consistent across alternative specifications, although statistical precision varies across subsamples and restrictions. We offer two complementary interpretations, while emphasizing that causal identification relies on the parallel trends and no‐interference assumptions.

Prices decline gradually after licenses are issued—about 0.19 SD on average. By contrast, consumption rises more slowly, by about 0.21 SD, with no immediate jump but a gradual, approximately linear increase thereafter. This asymmetry suggests that price reductions may alleviate some access barriers while other constraints on treatment uptake remain binding.

These differences between prices and consumption provide insights into the mechanisms of healthcare systems. The gradual price declines may be explained by almost‐immediate shifts in procurement and negotiations—especially when generic manufacturers are poised to enter the market—and by fast‐track approvals for lower‐cost versions. By contrast, the slower increase in consumption may be explained by logistical constraints that need to be overcome, scaling public procurement, ensuring regulatory approvals for generics, expanding storage, and addressing workforce bottlenecks. This highlights the role of health‐system capacity—including infrastructure, healthcare personnel, supply chains, and public awareness—which remains critical for translating cheaper drugs into treatment coverage (Macfarlane et al. 2000; Peters et al. 2008). Realizing the full public‐health benefits of compulsory licensing therefore requires complementary investments in delivery systems.

Even if the consumption response is moderate rather than transformative, the price reductions imply sizable fiscal gains. A decline of roughly 0.19 SD in standardised prices could translate into substantial savings for national health systems—ranging from about US$0.03 million in Ecuador to US$86.75 million in Thailand, and from US$61.07 million in Canada to US$120.82 million in Germany—under a homogeneous‐effect assumption. These potential savings highlight an additional function of licenses: containing public expenditure on essential medicines.

This heterogeneity matters for interpretation. Thailand accounts for 20 of the 39 implemented licenses in our sample, and the pooled ATT should therefore be read as an average that is substantially influenced by the Thai experience. Outside Thailand, point estimates remain directionally similar but are smaller and statistically imprecise.

A complete welfare assessment of compulsory licensing must also account for its potential dynamic effects on pharmaceutical innovation. The theoretical literature finds that the welfare effect is ambiguous: under some parameter configurations, the option of compulsory licensing can even strengthen R&D incentives by expanding markets otherwise unserved (Bennato and Valletti 2014). Empirical evidence on this dynamic channel remains scarce, and future research quantifying the impact on pharmaceutical R&D—particularly for neglected and emerging‐market diseases—would be a valuable complement to the access‐focused evidence presented here.

This study has several limitations that should be considered when interpreting the findings. First, identification relies on the parallel trends assumption, which is untestable in general. Although pre‐treatment event‐study estimates are broadly consistent with this assumption, 3 of 31 pre‐treatment CIs for consumption and 2 of 31 for prices exclude zero—primarily at the far edge of the pre‐treatment window where data become sparse. Compulsory licenses are not randomly assigned and may be triggered by time‐varying factors that could independently affect drug consumption and prices. Our covariate adjustment and sensitivity analyses mitigate but cannot fully resolve this concern.

Second, we focus on HIV because it has been central to global debates on access to medicines—particularly at the 2001 Doha Ministerial Conference—and because untreated HIV has high fatality and a relatively simple, once‐daily regimen (Zolopa 2010). These features make HIV a setting where the impact of compulsory licenses may be especially visible. However, diseases with different clinical and logistical profiles (e.g., multiple‐drug regimens, complex monitoring, cold‐chain requirements) may respond differently, limiting external validity beyond HIV.

Third, compulsory licenses may have coincided with increases in international development aid or donations for HIV treatment. In such cases, countries could receive substantial volumes of antiretroviral drugs via donor programs or external procurement. Because we rely on IQVIA data, which primarily captures sales and procurement through formal domestic channels, drug volumes supplied by external actors may be partially or wholly unobserved. Consequently, some access gains linked to aid may be misattributed or missed—especially on the consumption side. Triangulating IQVIA volumes against donor procurement records or UNAIDS treatment coverage estimates would help bound this source of bias, but lies beyond the scope of the present study.

Fourth, the study period also saw the rapid expansion of voluntary licensing agreements and the Medicines Patent Pool (MPP). These developments influence prices and availability independently of compulsory licenses, and our design does not permit clean separation of their effects. Including drug‐level indicators for voluntary license and MPP status as time‐varying covariates in future work would help isolate the incremental contribution of compulsory licensing.

Fifth, we cannot restrict the analysis to drug–country pairs with active patent protection at the time of the compulsory license because the TRIPS Flexibilities Database and the IQVIA patent‐status fields do not provide molecule–country–year patent expiry dates with sufficient precision. If some compulsory licenses were implemented when effective patent protection had already lapsed, the estimated price effect could be attenuated. At the same time, the TRIPS database records implemented rather than merely threatened licenses, suggesting that patent rights were still salient in many of these cases.

Finally, the IQVIA data available to us do not contain a reliable harmonized indicator separating originator from generic products at the molecule–country–year level. We therefore estimate the net association of compulsory licensing with aggregate commercial prices and volumes, rather than separately identifying generic entry, originator price responses, or substitution between them. Future work with more granular product‐level data could test the competition mechanism more directly.

These limitations are largely driven by data constraints and underscore the importance of interpreting our results in context. Future research with more comprehensive datasets—particularly those capturing low‐income countries, donor‐financed procurement, and off‐channel distribution—would paint a more complete picture of how compulsory licenses affect affordability and access. It would also be valuable to examine heterogeneity by therapeutic area and delivery complexity, and to better track dynamic responses over longer horizons.

## 5 Conclusions

Our findings are consistent with compulsory licensing being associated with improved commercial access to HIV medicines, conditional on the identifying assumptions. We find an estimated reduction in HIV drug prices—around 0.19 SD—and a corresponding estimated increase in drug consumption of about 0.21 SD. In the main specification, both estimates are statistically significant; across sensitivity analyses, the direction of the estimates is generally stable although precision varies. While not transformative, these estimated associations are economically meaningful, especially in resource‐constrained settings. In least‐developed countries, even moderate price reductions can yield substantial welfare gains by lowering healthcare costs and enabling more patients to initiate or maintain treatment.

Policy‐wise, the findings indicate that compulsory licenses can be a useful—though not sufficient—tool to improve patient access. They are associated with lower prices and can deliver sizable fiscal savings, but the full translation into expanded treatment coverage depends on the broader delivery context. Pairing compulsory licenses with targeted investments in health‐system capacity (procurement, regulation, supply chains, workforce, storage, and patient outreach) is more likely to unlock the full benefits—in terms of both expanded access and meaningful cost savings—than compulsory licenses alone. The impact of compulsory licenses should be further analyzed with more comprehensive datasets and for other therapeutic areas to understand their importance for patients within the global health policy toolkit, alongside system‐strengthening measures.

## Funding

This work was partially funded by the Swiss National Science Foundation.

## Conflicts of Interest

The authors declare no conflicts of interest.

## Supporting information


Supporting Information S1

## Data Availability

The data that support the findings of this study are available from IQVIA. Restrictions apply to the availability of these data, which were used under license for this study. Data are available from https://www.iqvia.com/ with the permission of IQVIA.
