# Supplementary material for: The Effects of Compulsory Licensing: A Case Study of HIV Drugs
Source: Health Econ. 2026 Jun 23;35(10):1427–34. doi: 10.1002/hec.70123 (PMC13532639; doi:10.1002/hec.70123)
Supplement: Supplementary file 1 [file HEC-35-1427-s001.pdf]

The effects of compulsory licensing: *a case study of HIV drugs*

Supplementary Material

Nicolau Martin-Bassols, Miquel Serra-Burriel, Kerstin Noelle Vokinger

Supplementary Table 1: Descriptive statistics for treated country-drug units (pre- and post-compulsory license)

Country	Molecule	Appr. year	ARV class	EML (year)	MPP (year)	Units pre	Units post	Price pre	Price post
Brazil	Efavirenz	1998	NNRTI	2002	2011	3,369	2,695	5.47	8.63
Canada	Lamivudine	1995	NRTI	2002	—	1,359,132	689,234	3.51	4.02
Canada	Nevirapine	1996	NNRTI	2002	—	432,073	424,921	3.64	3.32
Canada	Zidovudine	1987	NRTI	1993	—	424,283	207,785	1.46	2.57
China	Efavirenz	1998	NNRTI	2002	2011	30,782	43,707	0.84	1.47
China	Lopinavir / Ritonavir	2000	PI	2002	2011	172	83,360	1.03	0.69
Ecuador	Ritonavir	1996	PI	2002	—	3,305	542	1.64	1.63
Germany	Raltegravir	2007	INSTI	2015	2017	1,107,220	879,051	15.59	12.75
Indonesia	Lamivudine	1995	NRTI	2002	—	234,250	292,356	2.22	1.35
Indonesia	Nevirapine	1996	NNRTI	2002	—	47,684	224,044	0.24	0.24
Malaysia	Lamivudine/Zidovudine	1997	NRTI	2004	—	21,694	431,722	3.98	1.88
Malaysia	Zidovudine	1987	NRTI	1993	—	52,929	198,182	0.96	1.08
Pakistan	Lamivudine	1995	NRTI	2002	—	111,941	100,764	1.53	1.11
Philippines	Lamivudine	1995	NRTI	2002	—	160,478	96,628	2.26	3.21
Philippines	Lamivudine/Zidovudine	1997	NRTI	2004	—	711	160	4.61	4.53
Philippines	Nelfinavir	1997	PI	—	—	3,282	368	0.90	0.75
Philippines	Saquinavir	1995	PI	—	—	4,112	74	0.66	0.78
Philippines	Zidovudine	1987	NRTI	1993	—	662	0	0.72	0.71
Russia	Lenalidomide	2005	IMiD	—	—	42,198	211,047	420.04	132.07
Thailand	Abacavir	1998	NRTI	2002	2011	109,352	177,360	3.89	1.61
Thailand	Abacavir/Lamivudine	2004	NRTI	2010	2011	4,256	134,526	12.81	2.89
Thailand	Didanosine	1991	NRTI	2002	—	265,610	194,824	2.04	0.97
Thailand	Efavirenz	1998	NNRTI	2002	2011	716,550	1,800,347	1.22	0.59
Thailand	Emtricitabine/Tenofovir DF	2004	NRTI	2010	2011	232,707	787,337	1.59	0.89
Thailand	Indinavir	1996	PI	2002	—	281,841	96,840	0.32	0.22
Thailand	Lamivudine	1995	NRTI	2002	—	1,486,107	6,248,805	0.66	0.23
Thailand	Lam./Nev./Stav.	2001	NRTI+NNRTI	2004	—	2,109,525	1,133,884	0.40	0.20
Thailand	Lam./Nev./Zid.	2001	NRTI+NNRTI	2004	—	1,264,360	3,360,636	0.46	0.24
Thailand	Lamivudine/Stavudine	2001	NRTI	2004	—	109,747	214,697	0.24	0.17
Thailand	Lamivudine/Zidovudine	1997	NRTI	2004	—	296,658	1,006,060	1.25	0.49
Thailand	Lenalidomide	2005	IMiD	—	—	504	3,256	308.24	241.27
Thailand	Lopinavir/Ritonavir	2000	PI	2002	2011	262,636	2,197,242	1.90	0.92
Thailand	Nevirapine	1996	NNRTI	2002	—	295,558	1,377,645	0.84	0.23
Thailand	Raltegravir	2007	INSTI	2015	2017	7,890	99,904	6.97	5.59
Thailand	Ritonavir	1996	PI	2002	—	159,064	231,517	1.23	1.14
Thailand	Saquinavir	1995	PI	—	—	183,570	30,392	1.36	1.85
Thailand	Stavudine	1994	NRTI	2002	—	614,815	659,213	0.21	0.09
Thailand	Tenofovir DF	2001	NRTI	2005	2011	526,910	3,660,918	0.99	0.42
Thailand	Zidovudine	1987	NRTI	1993	—	967,345	2,615,568	0.25	0.13
Mean across treated units, excluding lenalidomide						375,475	802,792	2.43	1.88

Notes: Units are IQVIA standard units (one tablet/capsule/defined liquid volume). Price is list price per standard unit (USD). Pre = mean quarterly value in periods before compulsory license implementation; Post = mean quarterly value in periods on or after implementation. Appr. year = year of first FDA/EMA regulatory approval. ARV class: NRTI = nucleoside reverse transcriptase inhibitor; NNRTI = non-nucleoside RTI; PI = protease inhibitor; INSTI = integrase strand transfer inhibitor; IMiD = immunomodulatory drug (non-ARV). WHO EML = year first listed on the WHO Essential Medicines List; MPP = year Medicines Patent Pool license became active; '—' indicates not listed / no license as of 2022. Mean row excludes Lenalidomide (Russia and Thailand) to avoid distortion from the non-ARV outlier.

Supplementary Table 2: Pre-treatment parallel trends assessment: bootstrap 95% confidence intervals

Outcome	Pre-periods assessed	CIs including zero	Max $\lvert\hat{\alpha}_\tau\rvert$ (SD)	Post-ATT (SD)
Standardised units	31	28/31 (90.3%)	0.11	0.21
Standardised price	31	29/31 (93.5%)	$0.16^{\dagger}$	−0.19

Notes: Bootstrap 95% confidence intervals for pre-treatment event-study coefficients $\hat{\alpha}_\tau$ ($\tau = -31$ to -1) extracted from the main regression-adjusted specification (5,000 repetitions). Covariates: log GDP per capita (PPP), log population, HIV prevalence (GBD 2023), and drug age (years since first regulatory approval). The three consumption CIs excluding zero correspond to $\tau = \{-31, -8, -5\}$ with magnitudes of 0.11, 0.06, and 0.04 SD respectively—all smaller than the post-treatment ATT of 0.21 SD. The two price CIs excluding zero correspond to $\tau = \{-5, -2\}$ with magnitudes of 0.01 and 0.02 SD respectively—both well below the post-treatment ATT of −0.19 SD. †Largest pre-treatment price coefficient; its 95% CI is wide and includes zero.

Supplementary Table 3: Aggregate ATT estimates across all specifications

Specification	Consumption (std. units)			Price (std. price)		
	ATT	95% CI	p	ATT	95% CI	p
Main (with covariates)	0.21	[0.04, 0.38]	0.017	−0.19	[−0.36, −0.02]	0.027
No covariates	0.22	[0.06, 0.39]	0.007	−0.13	[−0.30, 0.03]	0.118
HQ countries	0.21	[0.03, 0.38]	0.021	−0.14	[−0.33, 0.05]	0.138
Not-yet-treated control	0.21	[0.03, 0.38]	0.019	−0.19	[−0.37, −0.02]	0.032
Thailand only	0.36	[0.09, 0.64]	0.010	−0.22	[−0.54, 0.09]	0.165
Excl. Thailand	0.10	[−0.08, 0.27]	0.267	−0.06	[−0.12, 0.01]	0.101
Same-molecule control	0.19	[0.01, 0.36]	0.037	−0.20	[−0.37, −0.04]	0.017
Anticipation 4 quarters	0.26	[0.08, 0.44]	0.004	−0.17	[−0.35, 0.00]	0.055
No fixed-dose combinations	0.19	[−0.01, 0.39]	0.060	−0.14	[−0.21, −0.06]	<0.001
No substitutes	0.25	[0.03, 0.48]	0.027	−0.20	[−0.44, 0.04]	0.101
Log outcomes	0.16	[−0.02, 0.34]	0.083	−0.32	[−0.48, −0.15]	<0.001

Notes: Each row reports the aggregate ATT for consumption (left) and price (right) under a different specification or sample restriction. 95% confidence intervals computed via 5,000 bootstrap repetitions, clustered at the molecule–country level. Values rounded to two decimal places. Main specification covariates: log GDP per capita (PPP), log population, HIV prevalence (GBD 2023), and drug age.

Supplementary Figure 1: Event study estimates, high-quality countries only (excl. Ecuador, Malaysia, Pakistan)

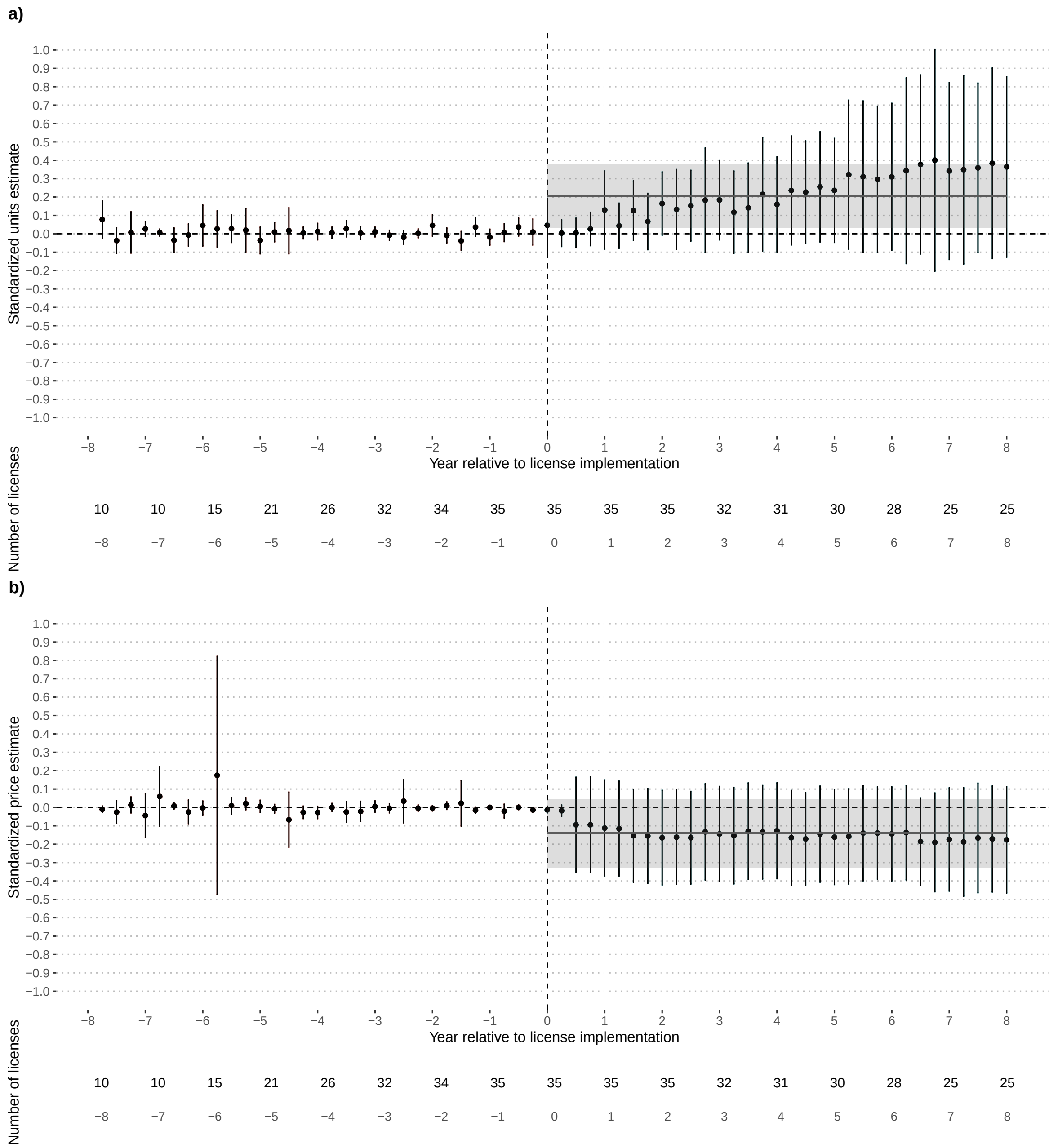

Notes: Countries with lower-quality IQVIA data excluded (Ecuador, Malaysia, Pakistan); 35 licenses retained. The control group is never treated. No covariate adjustment (sample restriction to high-quality countries removes the low-income treated units whose covariate variation is needed to identify the outcome regression). Clustered bootstrapped standard errors at the molecule–country level through 5,000 repetitions; 95% confidence intervals reported.

Supplementary Figure 2: Event study estimates, not-yet-treated control group

a)

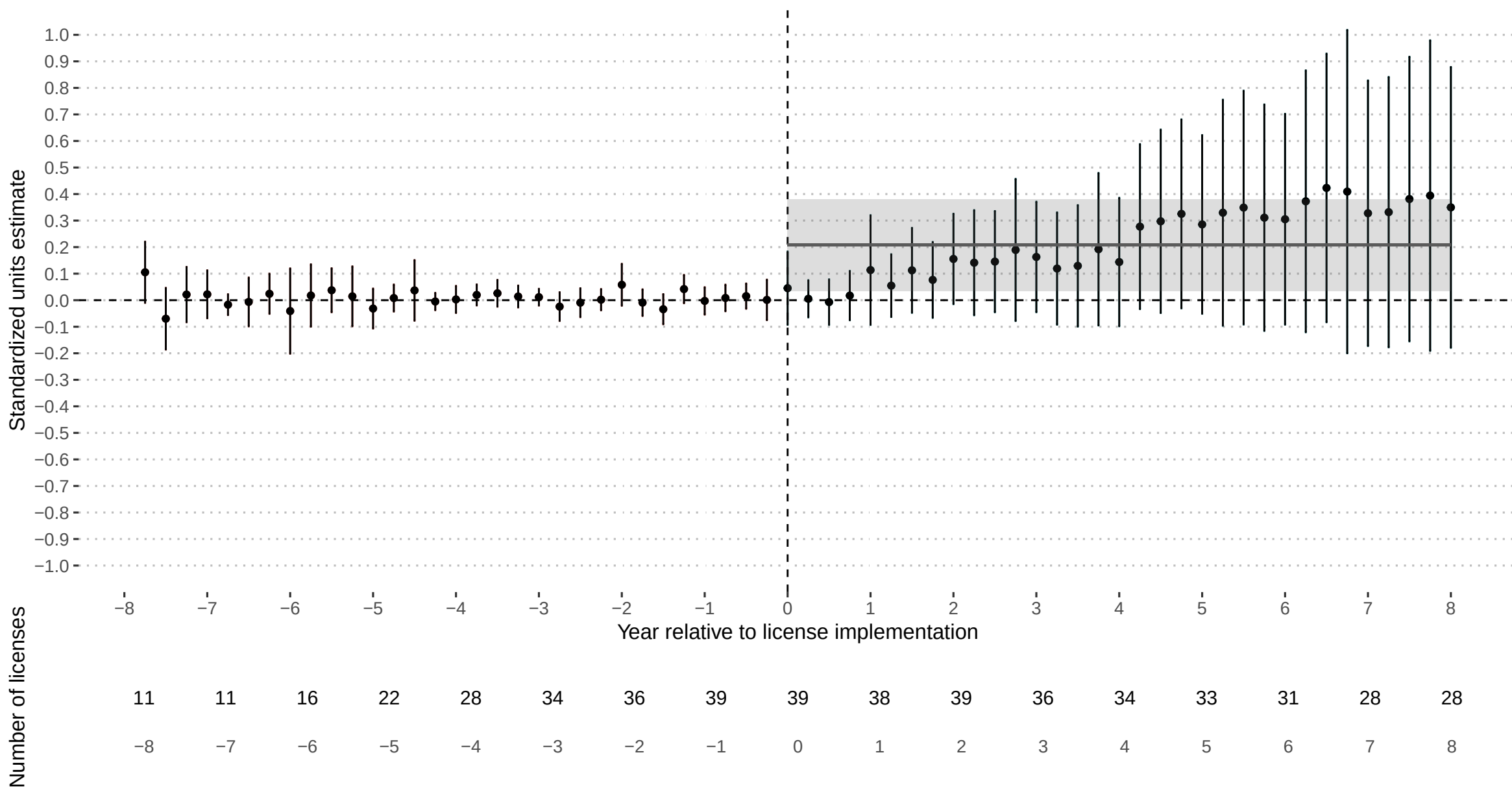

b)

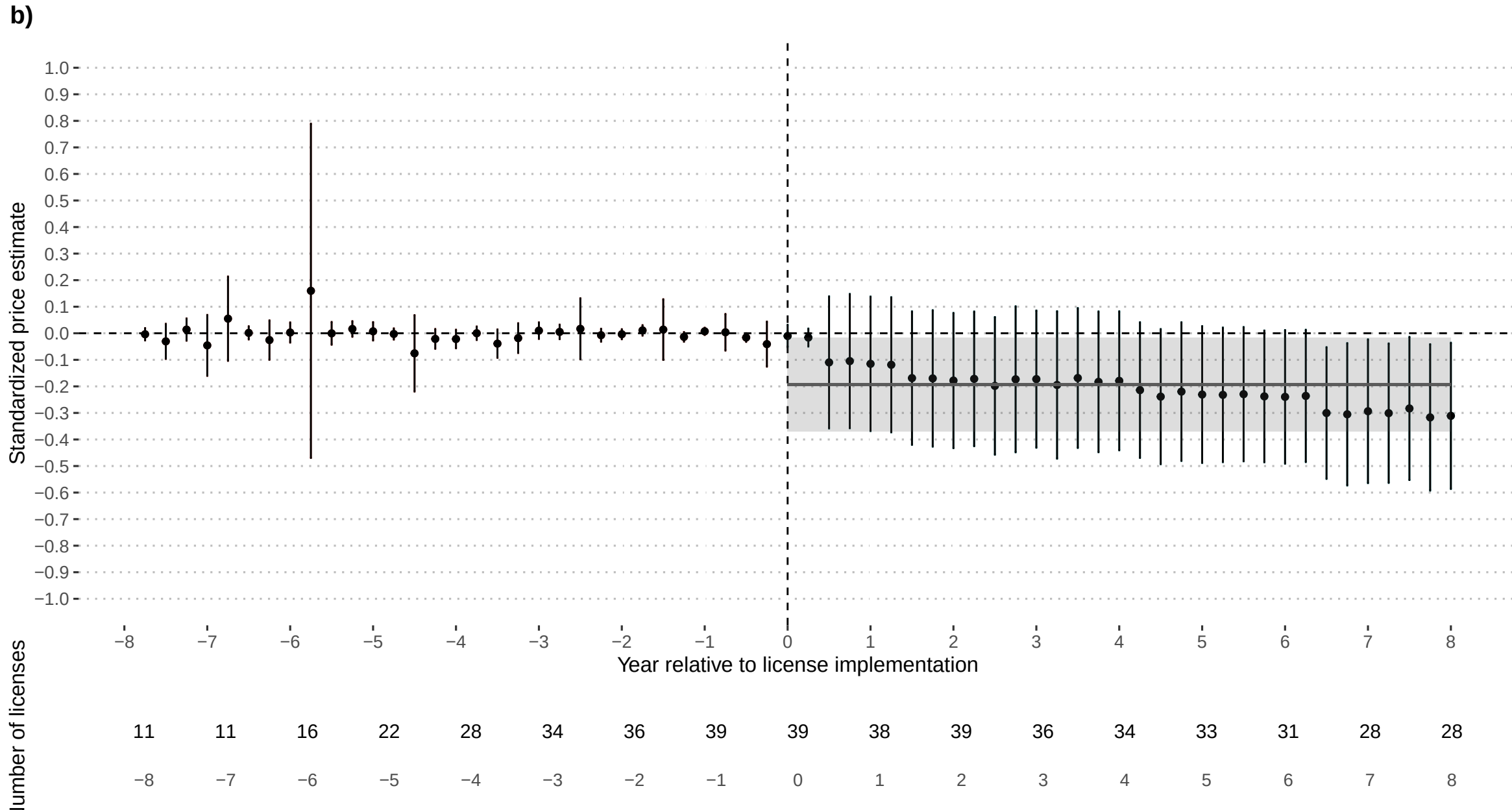

Notes: The control group consists of not-yet-treated country-drug units. Regression-adjusted Callaway–Sant'Anna estimator with covariates (log GDP per capita, log population, HIV prevalence, drug age). Clustered bootstrapped standard errors at the molecule–country level through 5,000 repetitions; 95% confidence intervals reported.

Supplementary Figure 3: Event study estimates, Thai market

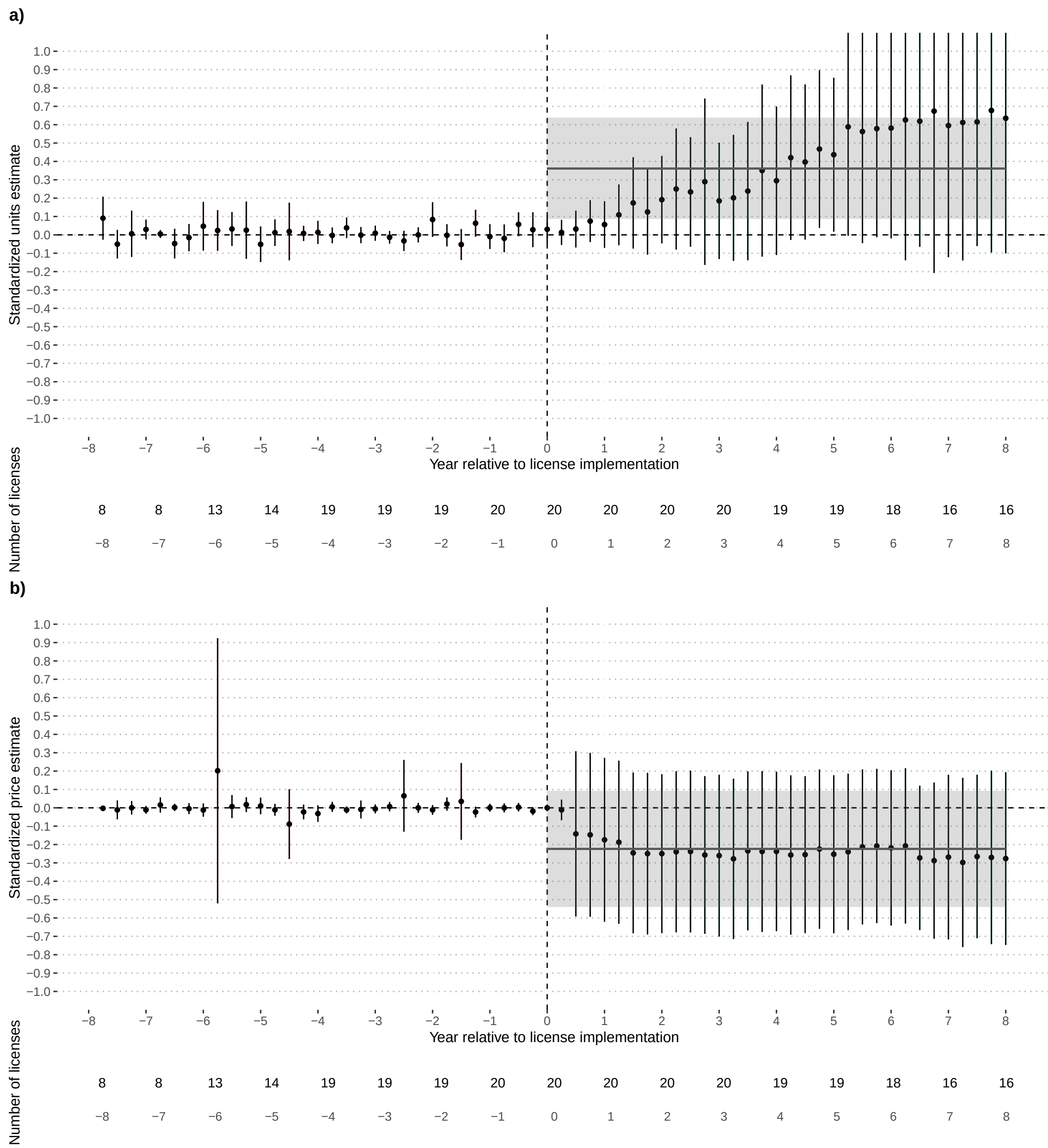

Notes: Sample restricted to Thailand (20 licenses) and never-treated control countries. No country-level covariates included (single treated country precludes identification of propensity score coefficients across countries). Clustered bootstrapped standard errors through 5,000 repetitions; 95% confidence intervals reported. The dotted vertical line marks the moment of compulsory license implementation. The solid horizontal line depicts the aggregated ATT, while the shaded area represents its 95% confidence interval.

Supplementary Figure 4: Event study estimates, excluding Thailand

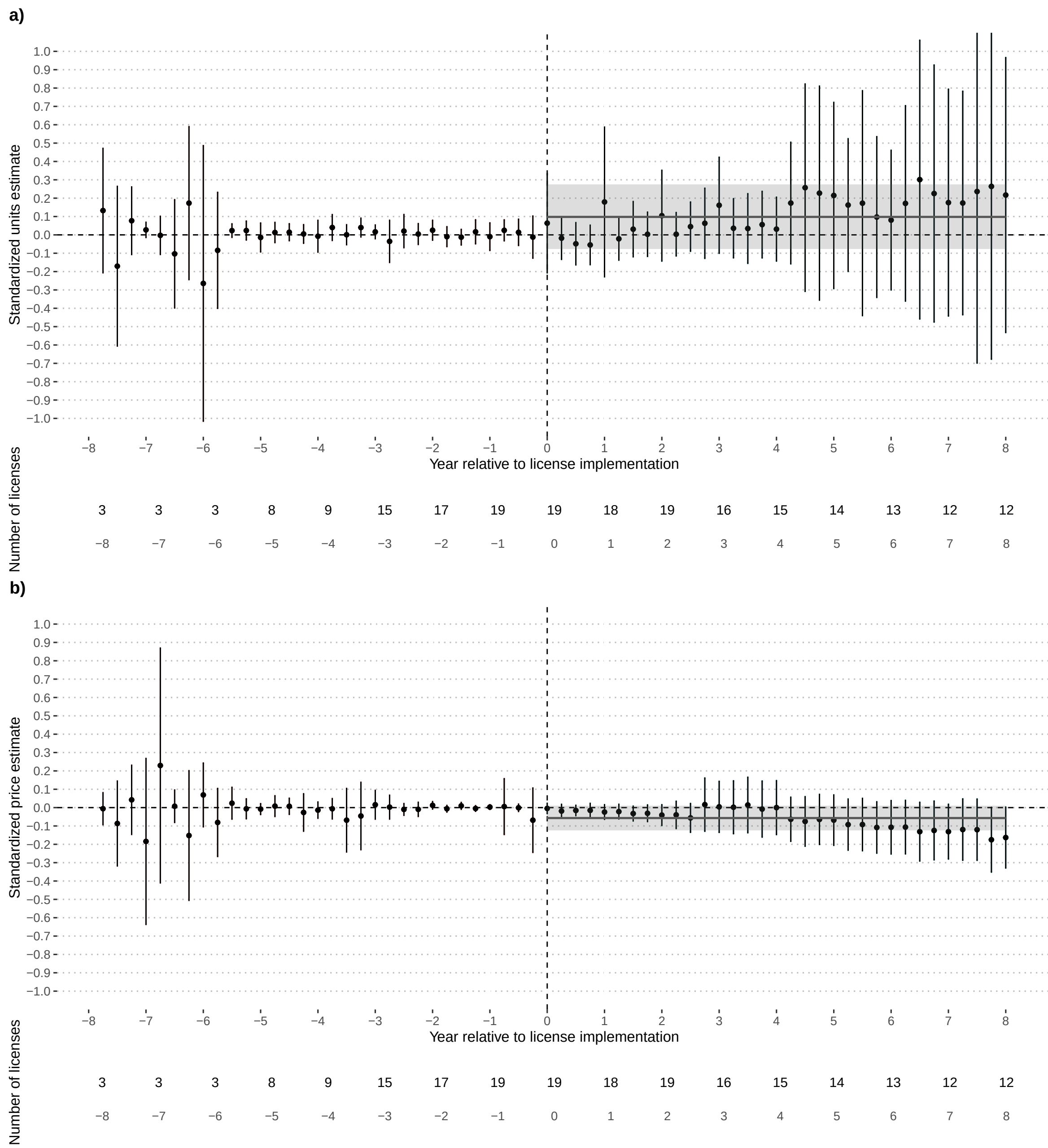

Notes: Thailand excluded from the estimation sample (19 licenses retained). The control group is never treated. Regression-adjusted Callaway–Sant'Anna estimator with covariates (log GDP per capita, log population, HIV prevalence, drug age). Clustered bootstrapped standard errors through 5,000 repetitions; 95% confidence intervals reported. The consumption ATT is 0.10 SD (95% CI: −0.08 to 0.27) and the price ATT is −0.06 SD (95% CI: −0.12 to 0.01).

Supplementary Figure 5: Event study estimates, same-molecule control group

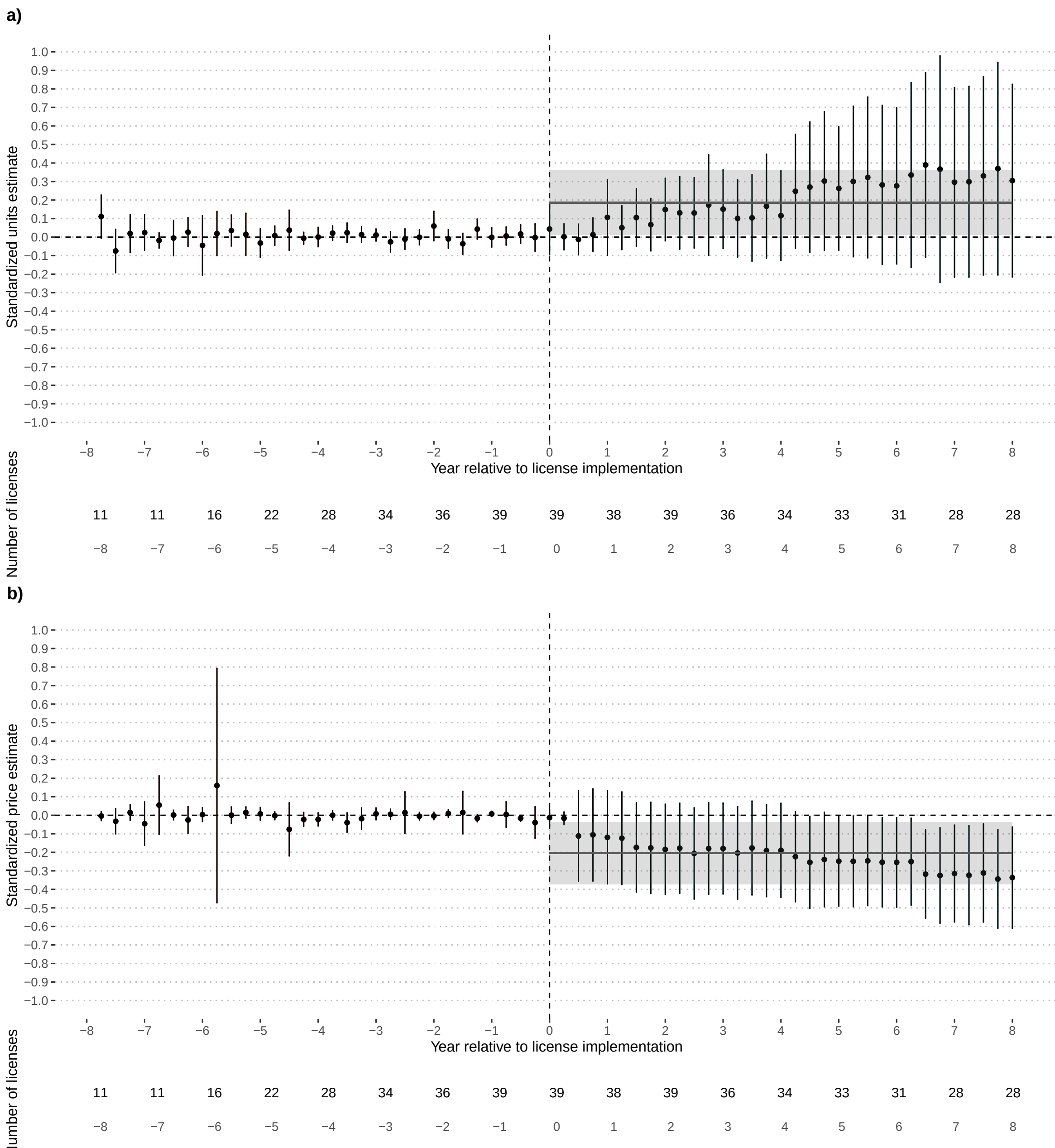

Notes: Control group restricted to the same molecules as those subject to a compulsory license, observed in countries that never issued a license for that molecule. Regression-adjusted Callaway–Sant'Anna estimator with covariates (log GDP per capita, log population, HIV prevalence, drug age). Clustered bootstrapped standard errors through 5,000 repetitions; 95% confidence intervals reported.

Supplementary Figure 6: Event study estimates, anticipation window of four quarters

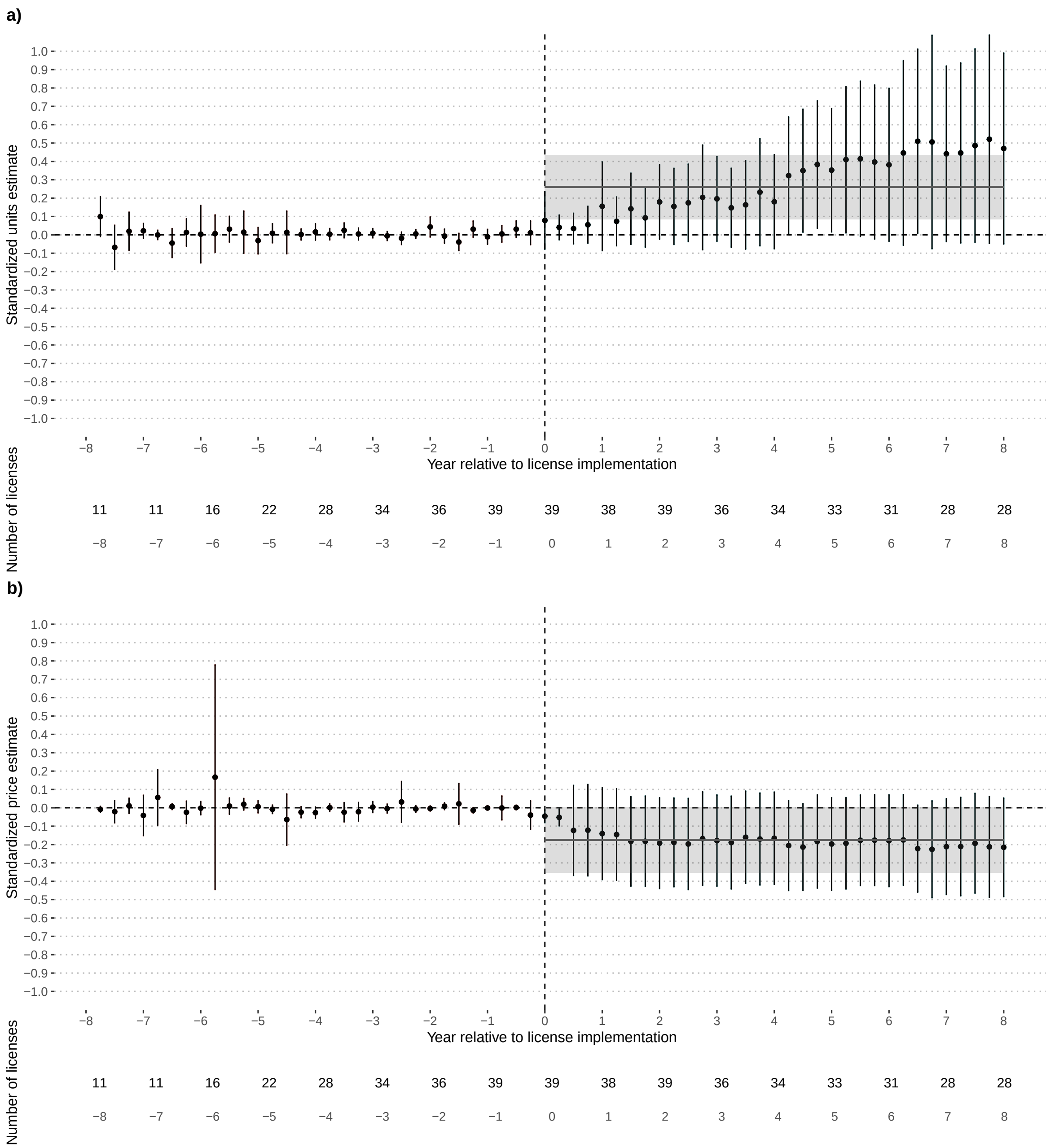

Notes: Anticipation window of four quarters (one year) included prior to the recorded implementation date, allowing for pre-announcement or negotiation-period effects. Control group: not-yet-treated. No covariates (shifted time window precludes standard covariate adjustment). Clustered bootstrapped standard errors through 5,000 repetitions; 95% confidence intervals reported. Consumption ATT: 0.26 SD (95% CI: 0.08 to 0.44). Price ATT: −0.17 SD (95% CI: −0.35 to 0.00).

Supplementary Figure 7: Event study estimates, single-ingredient molecules only

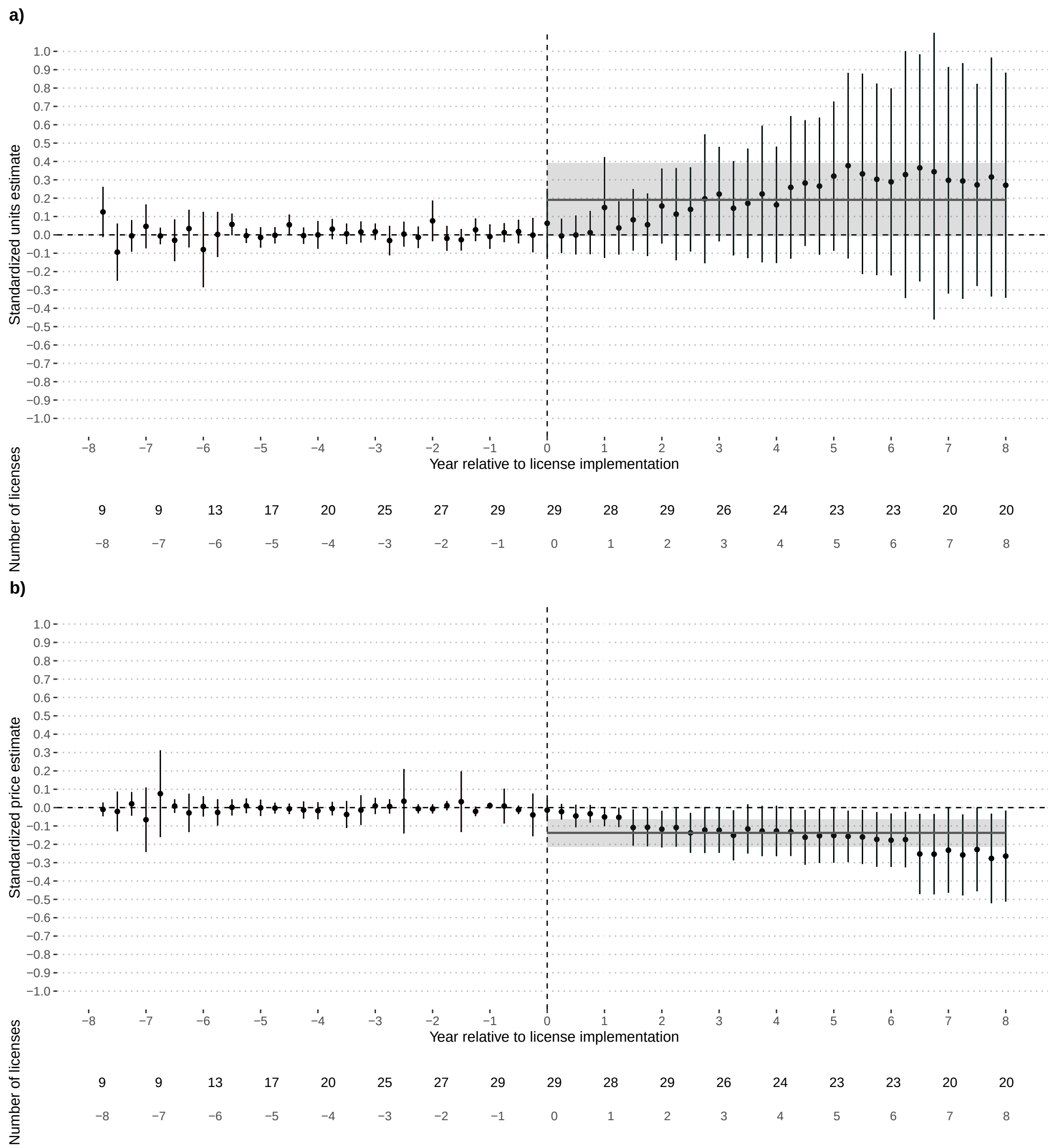

Notes: Fixed-dose combination products excluded; analysis restricted to 29 licenses for single-ingredient molecules. Control group: never treated. Regression-adjusted Callaway–Sant'Anna estimator with covariates (log GDP per capita, log population, HIV prevalence, drug age). Clustered bootstrapped standard errors through 5,000 repetitions; 95% confidence intervals reported.

Supplementary Figure 8: Event study estimates, excluding therapeutic substitutes

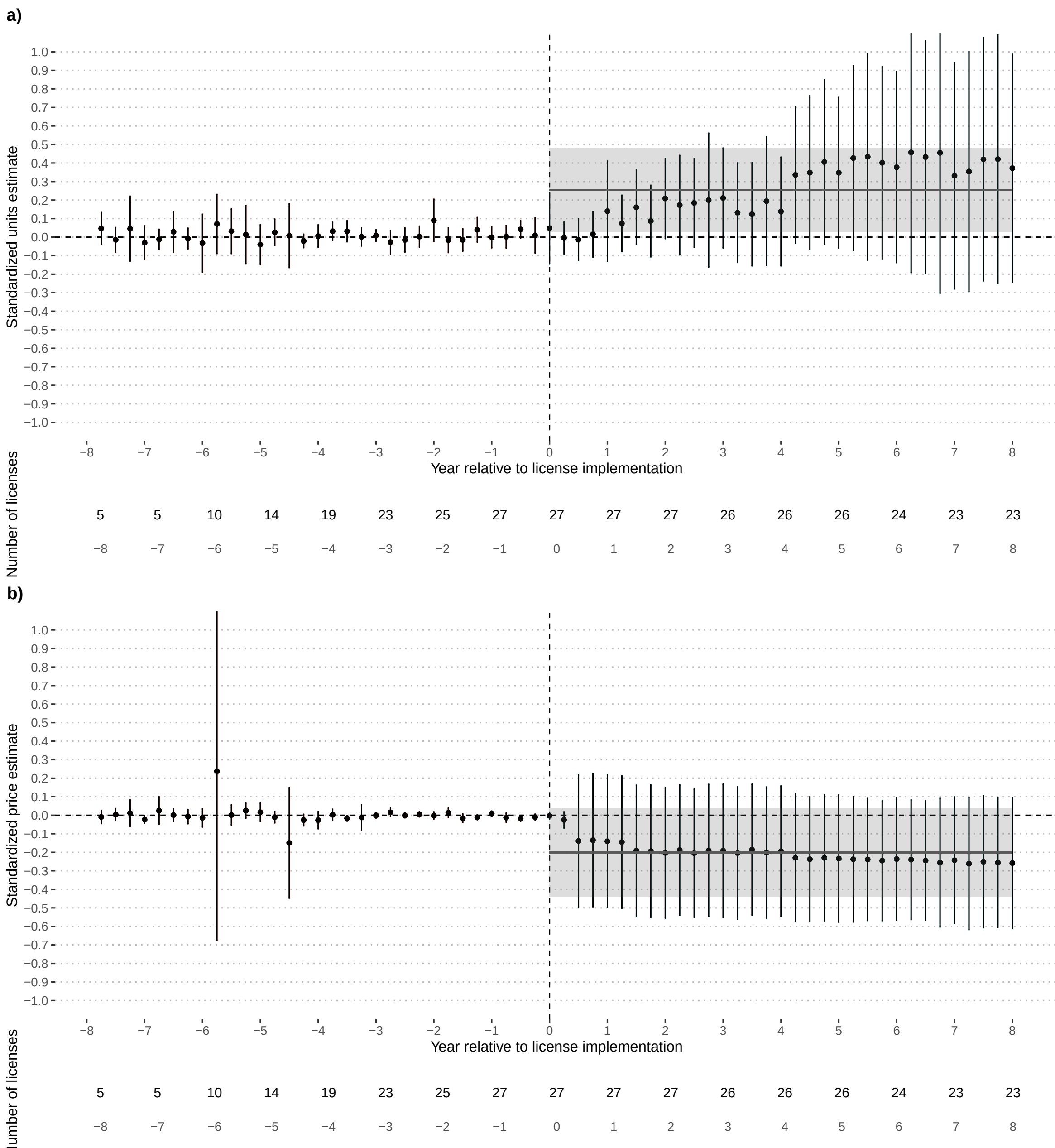

Notes: Protease inhibitors (Indinavir, Nelfinavir, Saquinavir, Lopinavir, Ritonavir), integrase inhibitors (Raltegravir), and non-ARV drugs (Lenalidomide) excluded. This sensitivity addresses the concern that CLs on one drug class may affect demand for substitutes in the control group. Control group: never treated. Clustered bootstrapped standard errors through 5,000 repetitions; 95% confidence intervals reported.

Supplementary Figure 9: Event study estimates, log-transformed outcomes

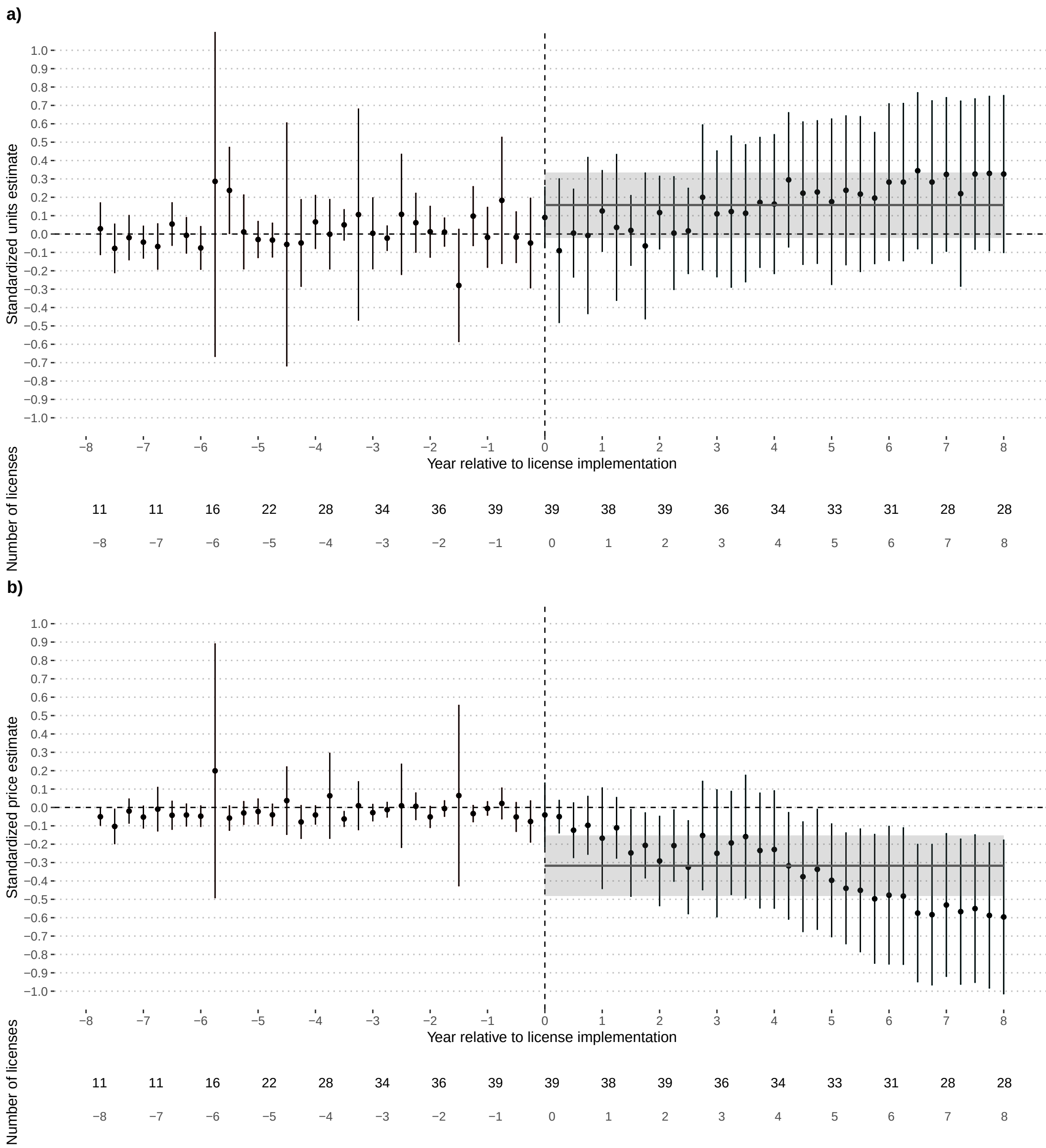

Notes: Outcomes are log(1 + raw value), normalised within country groups. Control group: never treated. Regression-adjusted Callaway–Sant'Anna estimator with covariates (log GDP per capita, log population, HIV prevalence, drug age). Clustered bootstrapped standard errors through 5,000 repetitions; 95% confidence intervals reported. The consumption ATT is 0.16 SD (95% CI: −0.02 to 0.34, $p = 0.083$) and the price ATT is −0.32 SD (95% CI: −0.48 to −0.15, $p < 0.001$), with the stronger price effect in logs reflecting the compressing effect of the log transformation on the right-skewed units distribution.

Supplementary Figure 10: Event study estimates, no covariate adjustment

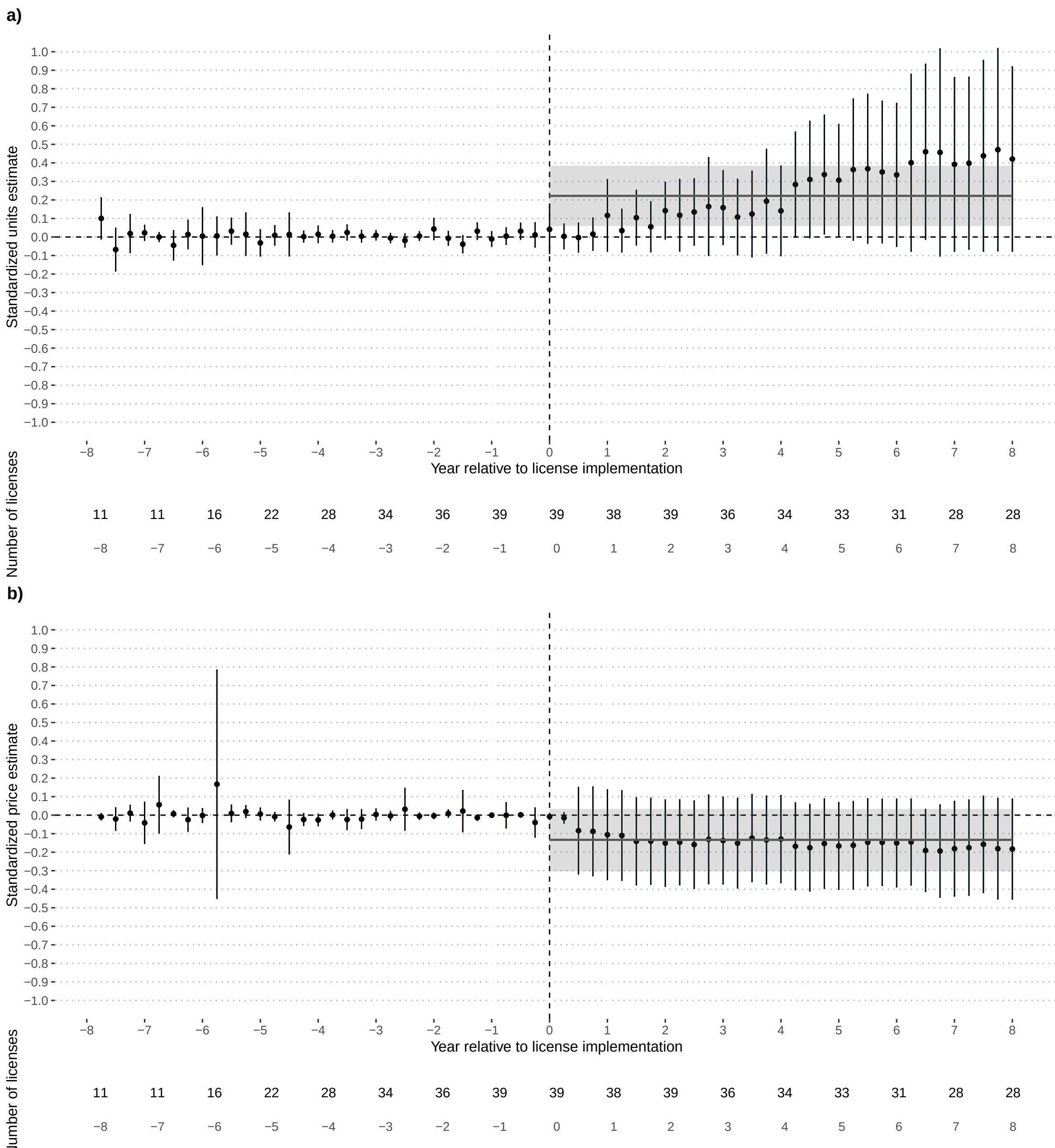

Notes: Main specification without covariate adjustment ($\mathbf{x} = 1$), replicating the original submission specification. Control group: never treated. Clustered bootstrapped standard errors through 5,000 repetitions; 95% confidence intervals reported. Consumption ATT: 0.22 SD (95% CI: 0.06 to 0.39); price ATT: −0.13 SD (95% CI: −0.30 to 0.03). Comparison with the main covariate-adjusted specification confirms that the stronger price effect in the main results is driven by the inclusion of covariates, particularly drug age.
